# Effects of acute and multi-day low-dose sodium bicarbonate intake on high-intensity endurance exercise performance in male recreational cyclists

**DOI:** 10.1007/s00421-024-05434-1

**Published:** 2024-02-29

**Authors:** S. Aktitiz, Ş. N. Koşar, H. H. Turnagöl

**Affiliations:** Faculty of Sport Sciences, Division of Exercise Nutrition and Metabolism, Hacettepe University, 06800 Ankara, Turkey

**Keywords:** Ergogenic aids, Alkalinizing, Buffering system, Anaerobic threshold, Time-to-exhaustion

## Abstract

**Purpose:**

This study aimed to compare the effects of acute and multi-day low-dose sodium bicarbonate (SB) intake on high-intensity endurance exercise performance.

**Methods:**

In a randomized, double-blind, cross-over design, twelve recreational male cyclists (age: 31.17 ± 4.91 years; $\dot{{\text{V}}}$O_2_peak: 47.98 ± 7.68 ml·kg^−1^·min^−1^) completed three endurance performance tests following acute SB (ASB, 0.2 g·kg^−1^ SB), multi-day SB (MSB, 0.2 g·kg^−1^·day^−1^ SB for four days), and placebo (PLA) intake. The high-intensity endurance performance was assessed with a cycling exercise test, wherein participants cycled on a bicycle ergometer at 95% of the predetermined anaerobic threshold for 30 min, followed by a time-to-exhaustion test at 110% of the anaerobic threshold. Data were analyzed using one-way and two-way repeated-measures ANOVA.

**Results:**

Significant main effects of supplementation protocol were evident in pre-exercise bicarbonate concentrations (*F* = 27.93; *p* < 0.01; partial eta squared (*η*^2^) = 0.72; false discovery rate (FDR)-adjusted *p* value = 0.001). Prior to performance test, blood bicarbonate concentrations were significantly higher in MSB (25.78 ± 1.63 mmol·L^−1 ^[95% CI 26.55–28.44] (*p* < 0.001; FDR-adjusted *p* value = 0.001)) and ASB (27.49 ± 1.49 mmol·L^−1^ [95% CI 24.75–26.81] (*p* < 0.001; FDR-adjusted *p* value = 0.007)) compared to PLA (23.75 ± 1.40 mmol·L^−1^ [95% CI 22.86 to 24.64]). Time-to-exhaustion increased in MSB (54.27 ± 9.20 min [95% CI 48.43–60.12]) compared to PLA (49.75 ± 10.80 min [95% CI 42.89–56.62]) (*p* = 0.048); however, this increase in MSB did not reach the significance threshold of 1% FDR (FDR-adjusted *p* value = 0.040). No significant difference was noted in exhaustion times between ASB (51.15 ± 8.39 min [95% CI 45.82–56.48]) and PLA (*p* > 0.05).

**Conclusion:**

Both acute and multi-day administration of low-dose SB improves buffering system in cyclists; nevertheless, neither intervention demonstrates sufficient efficacy in enhancing high-intensity endurance performance.

## Introduction

Sodium bicarbonate (SB) is the primary ergogenic aid that attenuates the accumulation of lactate and H^+^ ions (Freis et al. 2017; Krustrup et al. 2015) and thus improves sports performance, particularly in short-term high-intensity exercises based on glycolytic metabolism, such as sprinting, middle-distance running, and combat sports (Delextrat et al. 2018; Driller et al. 2012; Gough et al. 2017; Krustrup et al. 2015; Lopes-Silva et al. 2018; Marriott et al. 2015; Miller et al. 2016; Mueller et al. 2013). The majority of studies in the literature have focused on the effects of SB on sports activities lasting 1- to 7-min (Delextrat et al. 2018; Driller et al. 2012; Gough et al. 2017; Krustrup et al. 2015; Lopes-Silva et al. 2018; Marriott et al. 2015; Miller et al. 2016; Mueller et al. 2013). Additionally, there is a growing body of research investigating the potential benefits of SB on high-intensity endurance exercise, given the significant contribution of anaerobic glycolysis to overall energy production and the increased blood lactate concentrations observed, particularly in activities performed at or near the anaerobic threshold (Egger et al. 2014; Freis et al. 2017; Jones et al. 1977; Leach et al. 2023; McNaughton et al. 1999a, b; Potteiger et al. 1996; Stephens et al. 2002; Sutton, Jones, & Toews, 1981). Indeed, maintaining optimal pH concentrations throughout exercise is crucial for sustaining endurance performance at high intensities for prolonged periods of time, which contributes to enhance training capacity and adaptation. In this regard, SB supplementation may improve performance by enhancing the buffering system and facilitating the removal of H^ +^ ions, thereby maintaining pH during high-intensity endurance exercise performed at or near the anaerobic threshold, such as an hour cycling race at an anaerobic threshold of 4 mmol·L^−1^ (Bell, Furber, KA, Anton-Solanas, & Swart, 2017).

Studies showing that SB ingestion increases endurance performance have all examined the effects of acute ingestion (Dalle et al. 2021; Egger et al. 2014; George & MacLaren 1988; Jones et al. 1977; Leach et al. 2023; Sutton et al. 1981). The generally recommended acute dose of SB is 0.3 g·kg^−1^ which is typically consumed 90 min before exercise (Bishop et al. 2004; Brisola et al. 2015; Lopes-Silva et al. 2018; Marriott et al. 2015; Painelli Vde et al. 2013; Zabala et al. 2008). In addition, timing SB consumption in accordance with the time-to-peak alkalosis of the individual is a crucial factor that can augment its ergogenic effects (Boegman et al. 2020; Miller et al. 2016; Sparks et al. 2017). Nevertheless, in instances where determining the precise time-to-peak alkalosis is challenging, it is recommended that SB ingestion time be between 60 and 180 min before the commencement of exercise (Grgic et al. 2021). At these pre-exercise timings, recommended acute dose of SB (0.3 g·kg^−1^) has been shown to result in the greatest increase in blood pH and bicarbonate concentrations (Price & Singh, 2008) as well as a peak decrease in plasma H^+^ concentrations (Renfree, 2007). However, this dose has also been associated with increased salt intake (Siegler et al. 2012), which can cause gastrointestinal (GI) distress manifested by symptoms such as abdominal cramps, nausea, diarrhea, bloating, and vomiting (Durkalec-Michalski, Zawieja, Podgorski, Zawieja, et al., 2018a; Lancha Junior et al. 2015). Avoiding GI symptoms is especially crucial in endurance sports, as such symptoms can have a greater impact on performance in long-term exercise than in short-term exercise (Jeukendrup 2017). In recent years, researchers have investigated novel ingestion strategies to reduce or avoid the GI symptoms associated with the commonly recommended acute dose of SB. In this context, recent research suggests that acute intake of enteric formulated (Hilton et al. 2020a, b; Hilton et al. 2020a, b; Leach et al. 2023) or delayed release (Hilton et al. 2019) capsules could be effective in reducing the GI symptoms. Another strategy that has been studied is to use a lower dose taken on consecutive days, known as chronic or multi-day low dose (Durkalec-Michalski et al. 2018a, b; Durkalec-Michalski, Zawieja, Podgorski, Zawieja, et al., 2018a; Joyce et al. 2012). Limited studies of the multi-day low-dose SB ingestion has been shown to increase anaerobic performance and avoid GI symptoms (Durkalec-Michalski et al. 2018a, b). The daily doses used in these studies (Durkalec-Michalski et al. 2018a, b; Durkalec-Michalski, Zawieja, Podgorski, Zawieja, et al., 2018a; Joyce et al. 2012) varied from 0.025 to 0.5 g·kg^−1^, and the daily dose was either consumed all at once or divided into two to four doses over a period of 3 to 10 days. However, to date, no study has investigated the effects of multi-day low-dose SB ingestion on high-intensity endurance performance. The fact that some studies suggest improved exercise performance (George & MacLaren 1988; Gough et al. 2017) and an increase in blood bicarbonate concentration of 5.1–8.1 mmol·L^−1^ (Jones et al. 2016) even at an acute dose of 0.2 g·kg^−1^, and that 0.2 and 0.3 g·kg^−1^ SB ingestion result in similar increases in buffering capacity (Siegler et al. 2010), shows that 0.2 g·kg^-1^ of sodium bicarbonate is the highest dose that improves exercise performance without causing GI symptoms. Therefore, this study aimed to compare the effects of acute and multi-day low-dose SB (0.2 g.kg^−1^) ingestion on high-intensity endurance exercise performance in recreational male cyclists.

## Materials and methods

### Experimental approach to the problem

**Fig. 1:**
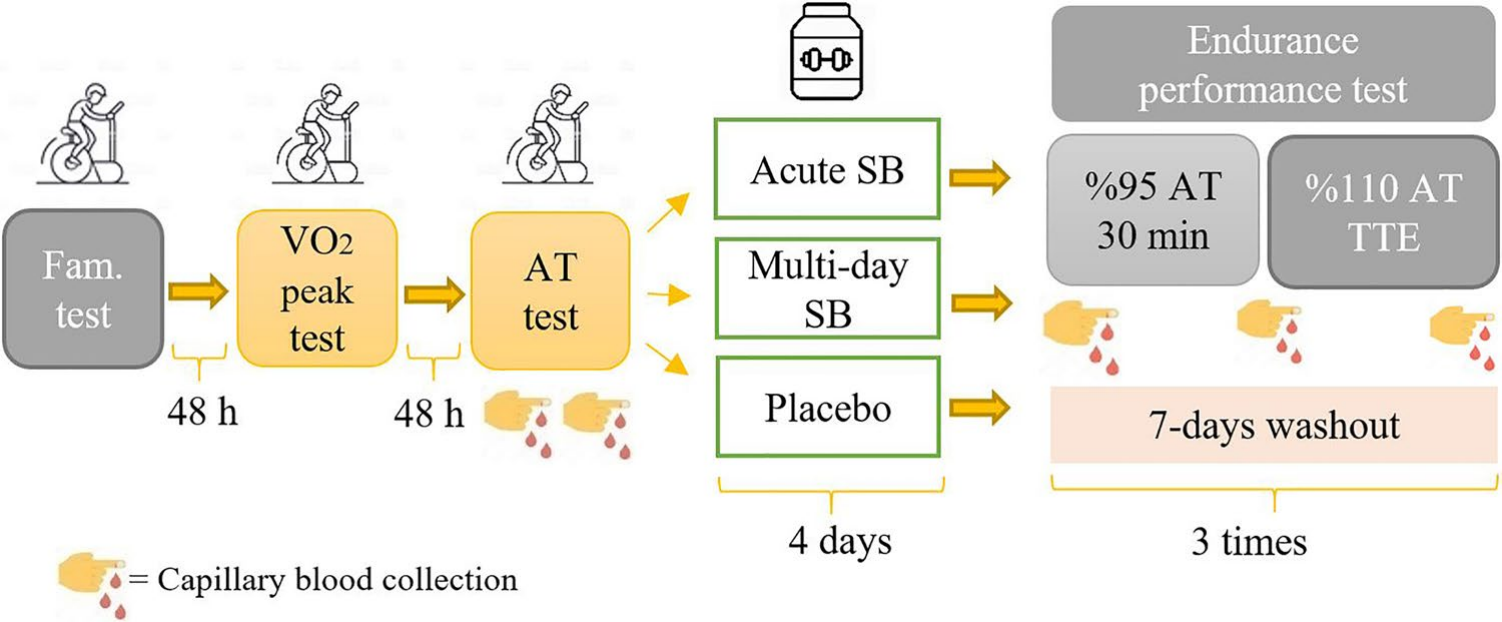
Study design. Fam. test: Familirization test; VO_2_peak test: Peak oxgen consumption test; AT test: Anaerobic threshold test; *SB* Sodium bicarbonate, *h* hours, *TTE* Time to exhaustion

This is a randomized, double-blind study with cross-over design. Each participant visited the laboratory six times (Fig. 1). During the first visit, body composition was measured using dual-energy X-ray absorptiometry (DXA) (Lunar Prodigy Pro Narrow Fan Beam (4.5°), GE Health Care, Madison Wisconsin, USA) following a 12-h fast. This visit also included familiarization tests to orient participants to the testing procedures of peak oxygen uptake ($\dot{{\text{V}}}$O_2_peak), anaerobic threshold, and time to exhaustion. During the second and third visits, participants underwent two incremental exercise tests on a cycle ergometer to determine their $\dot{{\text{V}}}$O_2_peak and anaerobic threshold, respectively. The first three visits were at least 48 h apart. On the 4th, 5th, and 6th visits, participants underwent an endurance performance test after consuming SB or placebo according to three different supplementation protocols in a randomized order with a 7-day washout period between the trials: multi-day SB (MSB), acute SB (ASB), and placebo (PLA). To determine blood lactate, pH, and bicarbonate concentrations, as well as partial oxygen pressure (pO_2_) and partial carbon dioxide pressure (pCO_2_), capillary blood samples were taken before, during (at the 30th minute), and immediately after the endurance performance tests performed following MSB, ASB, and PLA. In addition, GI symptoms were assessed at the end of the day (90 min after the second dose) during the 4-day supplementation protocol and immediately before the endurance exercise test.

### Participants

The sample size for repeated measures within factor analysis of variance (ANOVA) was determined using G*Power software (G*Power, version 3.1.9.2, Franz Faul, University of Kiel, Dusseldorf, Germany). The analysis indicated that 12 participants were required, with an α error probability of 0.05, a power of 0.80, and an effect size of 0.4. Thirteen participants, who met the inclusion criteria, volunteered to participate in this study, but one of them withdrew due to an injury sustained during training. Thus, this study involved 12 healthy male athletes (Mean ± standard deviation: body weight: 70.33 ± 7.53 kg, height: 1.76 ± 0.08 m, body fat percentage: 21.09 ± 4.79%) between the ages of 19 and 39 years (age = 31.17 ± 4.91 years) who regularly participated in cycling, duathlon, and triathlon training for at least two years (5.67 ± 3.79 years), with a minimum of three days per week (8.75 ± 4.43 h.week^−1^) (Table 1). Those with any known acute or chronic diseases or those using medication or supplements that could impact metabolism were excluded. Participants with a $\dot{{\text{V}}}$ O_2_peak less than 40 ml·kg^−1^·min^−1^ were also excluded. Prior to undergoing exercise testing, including $\dot{{\text{V}}}$O_2_peak, anaerobic threshold, and endurance performance, participants were requested to refrain from alcohol and caffeine for the last 24 h and from intense physical activity for the last 48 h. The study procedures were thoroughly explained both orally and in writing to the participants, who provided written consent before participating in this study. This study was approved by the Institutional Clinical Research Ethics Committee and was conducted in compliance with the Helsinki Declaration.

**Table 1:** Participants’ characteristics (*n* = 12)

	Mean ± SD	95% CI
Age (years)	31.17 ± 4.91	27.19–34.82
Experience in endurance sports (yrs)	5.67 ± 3.79	3.54–9.06
Training duration (h/wk)	8.75 ± 4.43	5.63–12.37
Body mass (kg)	70.33 ± 7.53	65.07–76.67
Height (cm)	176.3 ± 8.55	170.8–183.8
BMI (kg/m^2^)	22.57 ± 2.56	20.47–24.47
Body fat (%)	21.09 ± 4.79	17.00–24.34
Body fat mass (kg)	15.11 ± 4.18	11.70–18.19
Lean body mass (kg)	55.90 ± 5.32	49.96–57.66
Visceral fat mass (g)	379.6 ± 215.2	209.2–520.4
$\dot{V}$O_2peak_ (mL·kg^−1^·min^−1^)	47.98 ± 7.68	42.49–53.48
VO_2peak_ (L.min^−1^)	3.36 ± 0.40	3.08–3.65

*BMI* body mass index, $\dot{V}$*O*_*2peak*_ peak oxygen uptake, *CI* Confidence interval

### Procedures

*Supplementation protocol:* For the MSB protocol, participants were instructed to consume a total of 0.2 g·kg^−1^·day^−1^ SB for four consecutive days. This daily dose was divided into two equal parts, to be taken in the morning and evening. Each dose consisted of 0.1 g·kg^−1^ of SB dissolved in 200 ml of water and participants were required to consume it within a time frame of 15 min. For the PLA and ASB protocols, participants were instructed to consume a total daily dose of 0.025 g·kg^−1^ of placebo (table salt), divided into two equal parts dissolved in 200 ml of water. The divided doses were to be taken in the morning and in the evening for four consecutive days. After the four-day supplementation period, 90 min before the endurance performance test participants consumed 0.025 g·kg^−1^ placebo for the MSB and PLA protocols, while 0.2 g·kg^−1^ SB for the ASB protocol (Table 2). Due to budgetary constraints we were unable to determine individual time-to-peak blood bicarbonate concentration. Therefore, we decided to administer SB 90 min before exercise, which is the typical pre-exercise time period in the literature (Bishop et al. 2004; Brisola et al. 2015; Lopes-Silva et al. 2018; Marriott et al. 2015; Painelli Vde et al. 2013; Zabala et al. 2008).

**Table 2:** Different supplementation protocols

Day/Protocol	Multi-day SB	Acute SB	Placebo
Day 1	SB: 0.2 g·kg^−1^·day^−1^	PLA: 0.025 g·kg^−1^·day^−1^	PLA: 0.025 g·kg^−1^·day^−1^
Day 2	SB: 0.2 g·kg^−1^·day^−1^	PLA: 0.025 g·kg^−1^·day^−1^	PLA: 0.025 g·kg^−1^·day^−1^
Day 3	SB: 0.2 g·kg^−1^·day^−1^	PLA: 0.025 g·kg^−1^·day^−1^	PLA: 0.025 g·kg^−1^·day^−1^
Day 4	SB: 0.2 g·kg^−1^·day^−1^	PLA: 0.025 g·kg^−1^·day^−1^	PLA: 0.025 g·kg^−1^·day^−1^
Day 5: 90 min before the test	PLA: 0.025 g·kg^−1^·day^−1^	SB: 0.2 g·kg^−1^·day^−1^	PLA: 0.025 g·kg^−1^·day^−1^

*SB* Sodium bicarbonate, *PLA* Placebo

The supplements were prepared by an impartial researcher who was not involved in the performance test and data analysis process and given to the participants in a sealed bag. Participants were asked to take the supplement after dissolving it in 200 ml of water in accordance with the protocol. Thus, both the researchers conducting the exercise test and data analysis and the participants were kept unaware of the actual content of the supplements. Placebo and SB solutions were tested in an independent group. The similarity of appearance, taste, smell, texture, and palatability of the placebo (0.025 g-kg^−1^-day^−1^) and SB (0.2 g-kg^−1^-day^−1^) samples was rated by 10 testers as 4.4, 3.6, 4.4, 4.3, and 4.6 out of 5, respectively.

During the four-day supplementation period and endurance performance test days, GI symptoms were recorded using a validated questionnaire (Adam et al. 2005), which was administered at the end of each day (90 min after the second supplement) as well as immediately before the endurance exercise test. Participants rated the severity of 10 different GI symptoms (nausea, vomiting, bloating, abdominal cramps, early satiety, heartburn, sickness, loss of appetite, retrosternal discomfort, and upper abdominal pain) on a 10-point Likert-type scale ranging from 0 (no problem) to 10 (very severe problem).

*Diet monitoring:* To monitor their diet, the participants completed a 24-h food diary before each endurance performance test. Prior to the second and third endurance performance tests, they were asked to consume a diet similar to the diet before the first test. The food records were analyzed using the BeBis 6.1 Nutrition Information System (Dr J. Erhardt, Stuttgart, Hohenheim, Germany). Two hours prior to the endurance performance tests, participants were provided with a standardized breakfast of ~ 10 kcal.kg^−1^ composed of ~ 1.4 g.kg^−1^ of carbohydrates, ~ 0.3 g.kg^−1^ of fat, and ~ 0.45 g.kg^−1^ of protein (Ensure Plus, Abbott, USA). They were given the option to choose between two types of flour, strawberry or chocolate, and were instructed to consume it within a 15-min time frame. This product was chosen for its rapid digestion, moderate glycemic index, and ability to provide high energy without causing GI symptoms before exercise.

$\dot{V}$*O*_*2*_*peak test:* An incremental exercise test protocol was performed on a bicycle ergometer (COSMED E 200, Italy) to assess participants’ $\dot{{\text{V}}}$O_2_peak using breath-by-breath technology (Quark CPET, COSMED Cardio-Pulmonary Exercise Testing, Italy). The protocol included a 2-min warm-up period at an initial load of 60 W, followed by 30 W increments every 2 min. After the third stage (120 W), the load was increased every minute by 30 W. The highest value achieved over a 30-s sampling period was used as the $\dot{{\text{V}}}$O_2_peak value (Robergs et al. 2010). The test was terminated when at least two of the following criteria were met (Howley et al. 1995; Taylor et al. 1955):

1. Plateauing of oxygen uptake while increasing work rate, with an increase of no more than 2 ml·kg^−1^·min^−1^;
2. A respiratory exchange ratio greater than 1.1;
3. Heart rate within 10 beats·min^−1^ of the predicted maximum (220-age); and
4. A score of at least 18 on the 6–20 Borg’s rate of perceived exertion (RPE).

The formula used to calculate peak power output (PPO) was as follows (Jeukendrup et al. 1996):

$$PPO={W}_{com}+30\frac{t}{60},$$

where:

- ${W}_{com}$ Represents the power output (in watts) of the last completed workload,
- *t* is the time (in seconds) completed during the final incomplete workload,
- 60 denotes the duration (in seconds) of each workload increment, and
- 30 represents the workload increment.

*Determination of anaerobic threshold:* To determine the individual anaerobic threshold of the participants, an incremental exercise test protocol was employed using a bicycle ergometer (COSMED E 200, Italy). The protocol included a 3-min warm-up period at an initial load of 50 W, followed by 25 W increments every 3 min which was adapted from Eggers et al. (2014). Blood samples were collected from the fingertips of the participants at the beginning, at the end of each 3-min period, and immediately after exhaustion to measure lactate concentrations using a lactate analyzer (ABL 9 Blood Gas Analyzer, Radiometer, Brønshøj, Denmark). Individual anaerobic threshold was determined according to the onset of blood lactate accumulation (OBLA; 4 mmol·L^−1^) using the Lactate-e software (Newell et al. 2007).

*Endurance performance test:* A two-stage endurance performance test on a bicycle ergometer (COSMED E 200, Italy) was conducted to determine the effect of bicarbonate consumption on long-term high-intensity exercise performance, 90 min after SB or placebo intake. The test began with a 3-min warm-up period at a workload of 50 W. In the first phase of the test, the participants pedaled at 70 rpm for 30 min at 95% of their individual AT. The second phase of the test commenced immediately after the completion of this stage. During this stage, the workload was increased to 110% of the individual AT, and participants continued pedaling at 70 rpm until exhaustion (Egger et al. 2014). Time to exhaustion was recorded and used as the endurance exercise performance. No encouragement method was used to motivate participants during the test. Fingertip capillary blood samples were collected at rest, at the 30th minute of exercise, and at exhaustion to determine blood pH, lactate, and bicarbonate concentrations by using a blood gas analyzer (ABL 9 Blood Gas Analyzer, Radiometer, Brønshøj, Denmark). Heart rate was recorded using a heart rate monitor (Polar RS800, Finland). No verbal encouragement was given to the participants during the tests. All endurance performance tests were performed at the same time of the day (07:30–09:30 in the morning) to avoid circadian rhythm effects. Each volunteer was ensured to take the 3 performance tests performed on different days at the same time of the day.

### Statistical analyses

The normal distribution of the data was assessed using the Shapiro–Wilk test. One-way repeated-measures ANOVA was used to determine the main effects of supplementation protocols on exhaustion time and pre-exercise blood pH, lactate, and bicarbonate concentrations. Two-way repeated-measures ANOVA was used to determine the main effects of supplementation protocol (MSB, ASB, and PLA) and time (pre-exercise, 30th min and immediately after exercise) on blood pH, bicarbonate, lactate, pCO_2_, pO_2_, and heart rate. The Mauchly’s test was used to examine the assumption of sphericity. If the sphericity assumption was violated, the degrees of freedom were corrected using the Greenhouse–Geisser estimates of sphericity. Pairwise comparisons were performed using the Bonferroni post hoc test. Effect sizes (ES) were calculated as partial eta squared (*η*_p_^2^) (Lakens 2013) and were interpreted as follows: *η*_p_^2^ ≤ 0.01 small impact; *η*_p_^2^ ≤ 0.06 medium impact; and *η*_p_^2^ ≤ 0.14 large impact (Hopkins et al. 2009). A Benjamini–Hochberg false discovery rate (FDR, Q) of < 1% was used to analyze all the *p* values (GraphPad Prism version 8.4.3; GraphPad Software, La Jolla, California, USA) and *p* values equal or higher than 0.01 were considered “false discoveries” (Benjamini & Hochberg 1995). All data are presented as mean ± SD as well as 95% confidence interval (95% CI). Statistical analyses were performed using SPSS (version 23.0; IBM Corp., Armonk, NY, USA), and the significance level was set at *p* < 0.05.

## Results

### Blood gas analyses

**Fig. 2:**
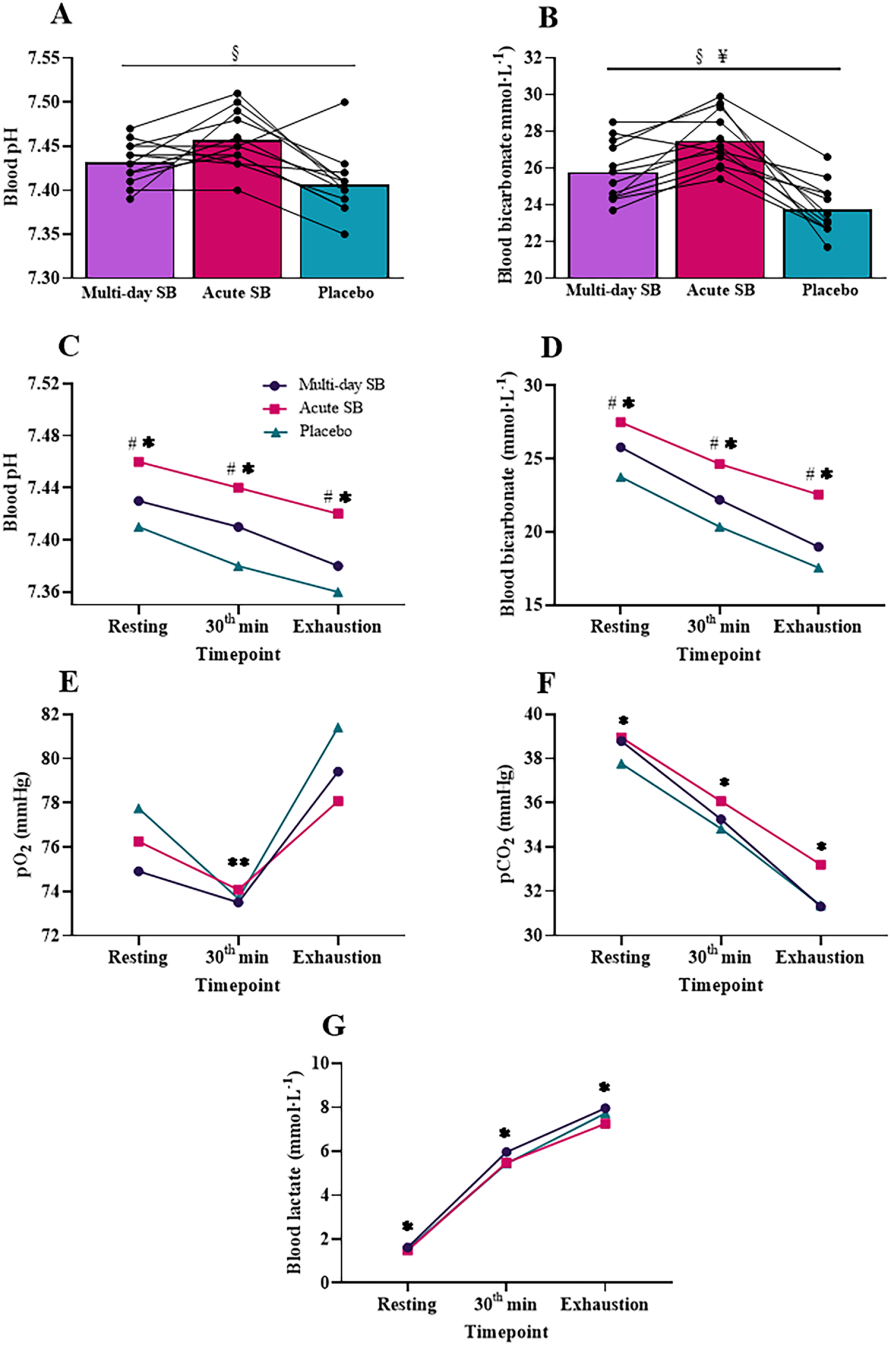
Blood gas parameters following different bicarbonate interventions (*n* = 12). §: Significant difference between ASB and PLA (*p* < 0.05); ¥: Significant difference between ASB, MSB, and PLA across all pairwise comparisons (*p* < 0.05); #: Significant difference between all bicarbonate interventions (*p* < 0.05); *: Significant difference between all time points (*p* < 0.05); **: Significant difference between the values at 30th minute and at the time of exhaustion (*p* < 0.05)

Significant main effects of supplementation protocol were evident in pre-exercise blood pH (*F* = 10.49; *p* < 0.01; *η*^2^ = 0.49; FDR-adjusted *p* value = 0.001) and bicarbonate concentrations (*F* = 27.93; *p* < 0.01; *η*^2^ = 0.72; FDR-adjusted *p* value = 0.001) (Fig. 2A and B, respectively). Although pre-exercise blood pH concentrations in ASB (7.46 ± 0.03 [95% CI 7.42– 7.45]) and MSB (7.43 ± 0.02 [95% CI 7.44– 7.48]) were higher compared to PLA (7.41 ± 0.036 [95% CI 7.38– 7.43]), only the difference between ASB and PLA reached a statistically significance concentration (*p* < 0.01 FDR-adjusted *p* value = 0.004). Statistically no significant difference was found between ASB and MSB in pre-exercise blood pH concentrations (*p* > 0.05). Pre-exercise blood bicarbonate concentrations were significantly higher following ASB (27.49 ± 1.49 mmol·L^−1^ [95% CI 24.75–26.81]) (*p* < 0.001; FDR-adjusted *p* value = 0.007) and MSB (25.78 ± 1.63 mmol·L^−1^ [95% CI 26.55–28.44]) (*p* < 0.01; FDR-adjusted *p* value = 0.000) compared to PLA (23.75 ± 1.40 mmol·L^−1^ [95% CI 22.86–24.64]). In addition, pre-exercise blood bicarbonate concentrations in ASB were significantly higher compared to MSB (*p* < 0.01; FDR-adjusted *p* value = 0.006). Pre-exercise lactate concentrations were similar between MSB (1.62 ± 0.15 mmol·L^−1^ [95% CI 1.29–1.95]), ASB (1.48 ± 0.12 mmol·L^−1^ [95% CI 1.21–1.74]), and PLA (1.54 ± 0.10 mmol·L^−1^ [95% CI 1.32–1.77]) (*p* > 0.05).

Significant main effects of supplementation protocol and exercise were observed on blood pH (Supplementation protocol: *F* = 37.92; *p* < 0.001; *η*^2^ = 0.78; FDR-adjusted *p* value = 0.000; Exercise: *F* = 12.74; *p* < 0.001; *η*^2^ = 0.54; FDR-adjusted *p* value = 0.000) and bicarbonate concentrations (Supplementation protocol: *F* = 45.18; *p* < 0.001; *η*^2^ = 0.80; FDR-adjusted *p* value = 0.000; Exercise: *F* = 43.91; *p* < 0.001; *η*^2^ = 0.80; FDR-adjusted *p* value = 0.000), with no significant interaction effect of protocol and exercise (blood pH: *F* = 0.63; *p* > 0.05; *η*^2^ = 0.05; blood bicarbonate: *F* = 1.64; *p* > 0.05; *η*^2^ = 0.13). Bonferroni post hoc analysis indicated that blood pH and bicarbonate concentrations were significantly different across the supplementation protocols, with ASB > MSB > PLA (*p* < 0.05). Exercise significantly reduced blood pH and bicarbonate concentrations at all time periods with pre-exercise > 30th minute of exercise > exhaustion time, according to Bonferroni post hoc analysis (*p* < 0.05) (Table 3, Fig. 2C and D, respectively).

**Table 3:** Blood pH, lactate, bicarbonate, pO_2_, and pCO_2_ levels measured before exercise, at the 30th minute of exercise, and at the time of exhaustion during the endurance performance test for sodium bicarbonate and placebo supplementation protocols (*n* = 12)

	MSB	ASB	PLA	P_prot_	P_time_	P_protxtime_
pH						
Pre-exercise	7.43 ± 0.022 (7.42–7.44)	7.46 ± 0.033 (7.43–7.48)	7.41 ± 0.036 (7.38–7.43)			
30th minute	7.41 ± 0.024 (7.39–7.42)	7.44 ± 0.033 (7.42–7.46)	7.38 ± 0.032 (7.36–7.40)	0.000^#^	0.000*	0.646
Exhaustion time	7.38 ± 0.047 (7.39–7.42)	7.42 ± 0.051 (7.39–7.46)	7.36 ± 0.041 (7.33–7.39)			
Lactate (mmol·L^−1^)						
Pre-exercise	1.62 ± 0.517 (1.29–1.95)	1.48 ± 0.420 (1.21–1.74)	1.54 ± 0.353 (1.32–1.77)			
30th minute	5.96 ± 2.16 (4.59–7.33)	5.48 ± 1.32 (4.65–6.32)	5.44 ± 1.20 (4.68–6.20)	0.378	0.000*	0.829
Exhaustion time	7.96 ± 2.51 (6.36–9.56)	7.25 ± 1.72 (6.16–8.34)	7.73 ± 2.41 (6.21–9.26)			
Bicarbonate (mmol·L^−1^)						
Pre-exercise	25.78 ± 1.63 (24.75–26.82)	27.49 ± 1.49 (26.55–28.44)	23.75 ± 1.40 (22.86–24.64)			
30th minute	22.20 ± 1.73 (21.10–23.30)	24.65 ± 2.34 (23.17–26.13)	20.35 ± 1.59 (19.34–21.36)	0.000^#^	0.000*	0.182
Exhaustion time	18.98 ± 2.00 (17.71–20.26)	22.55 ± 3.73 (20.18–24.92)	17.56 ± 2.66 (15.87–19.25)			
pO_2_ (mm Hg)						
Pre-exercise	74.91 ± 6.63 (70.71–79.13)	76.25 ± 6.96 (71.83–80.67)	77.75 ± 8.51 (72.34–83.16)			
30th minute	73.50 ± 6.56 (69.33–77.67)	74.08 ± 6.02 (70.26–77.91)	73.67 ± 4.69 (70.68–76.65)	0.401	0.002**	0.848
Exhaustion time	79.41 ± 8.10 (74.27–84.56)	78.08 ± 8.13 (72.91–83.25)	81.41 ± 4.50 (78.56–84.28)			
pCO_2_ (mm Hg)						
Pre-exercise	38.79 ± 2.23 (37.38–40.26)	38.95 ± 2.36 (37.45–40.46)	37.77 ± 2.71 (36.05–39.49)			
30th minute	35.25 ± 2.62 (33.59–36.92)	36.07 ± 2.37 (34.04–38.10)	34.83 ± 2.44 (33.28–36.37)	0.035	0.000*	0.652
Exhaustion time	31.31 ± 2.71 (29.58–33.03)	33.20 ± 3.50 (30.98–35.42)	31.35 ± 2.83 (29.56–33.16)			

*pO*_*2*_ partial pressure of oxygen, *pCO*_*2*_ Partial pressure of carbon dioxide, *p*_*prot*_ Difference due to supplemetation protocol, p_time_ Difference due to exercise phase, *p*_*prot x time*_ supplemetation protocol x time interaction, Values are mean ± SD (95% confidence interval) for 12 participants

#Significantly different across supplemetation protocols *p* < 0.05

*Significantly different across exercise phases *p* < 0.05

**Significantly different between the 30th minute of exercise and the time of exhaustion *p* < 0.05

Significant main effects of exercise were found on blood lactate (F = 102.43;* p* < 0.001; *η*^2^ = 0.90; FDR-adjusted *p* value = 0.000), pO_2_ (*F* = 8.13; *p* < 0.01; *η*^2^ = 0.43; FDR-adjusted *p* value = 0.002), and pCO_2_ (*F* = 38.97; *p* < 0.001; *η*^2^ = 0.78; FDR-adjusted *p* value = 0.000). Exercise significantly increased blood lactate concentrations at all time periods with pre-exercise < 30th minute of exercise < exhaustion time, according to Bonferroni post hoc analysis (*p* < 0.05) (Table 3, Fig. 2G). The post hoc analysis showed that 30 min of exercise did not change pO_2_ values compared to pre-exercise values (*p* > 0.05); however, pO_2_ values at the time of exhaustion were significantly higher compared to values at 30th minute of exercise (*p* < 0.05) (Table 3, Fig. 2E). No significant main effect of supplementation protocol (blood lactate: *F* = 1.02; *p* > 0.05; *η*^2^ = 0.09; pO_2_: *F* = 0.95; *p* > 0.05; *η*^2^ = 0.08; *F* = 3.94; *p* < 0.05; *η*^2^ = 0.26; FDR-adjusted *p* value = 0.032) or interaction effect of protocol and exercise (blood lactate: *F* = 0.37; *p* > 0.05; *η*^2^ = 0.03; pO_2_: *F* = 0.34; *p* > 0.05; *η*^2^ = 0.03; pCO_2_: *F* = 0.62; *p* > 0.05; *η*^2^ = 0.05)) was found on blood lactate, pO_2_, or pCO_2_. Although two-way repeated measure ANOVA revealed a significant main effect of supplementation protocol on pCO_2_ values, both Bonferroni post hoc analysis and FDR-adjusted *p* value = 0.032 indicated that this was a false discovery.

### Exhaustion times and heart rate values during endurance performance test

**Fig. 3:**
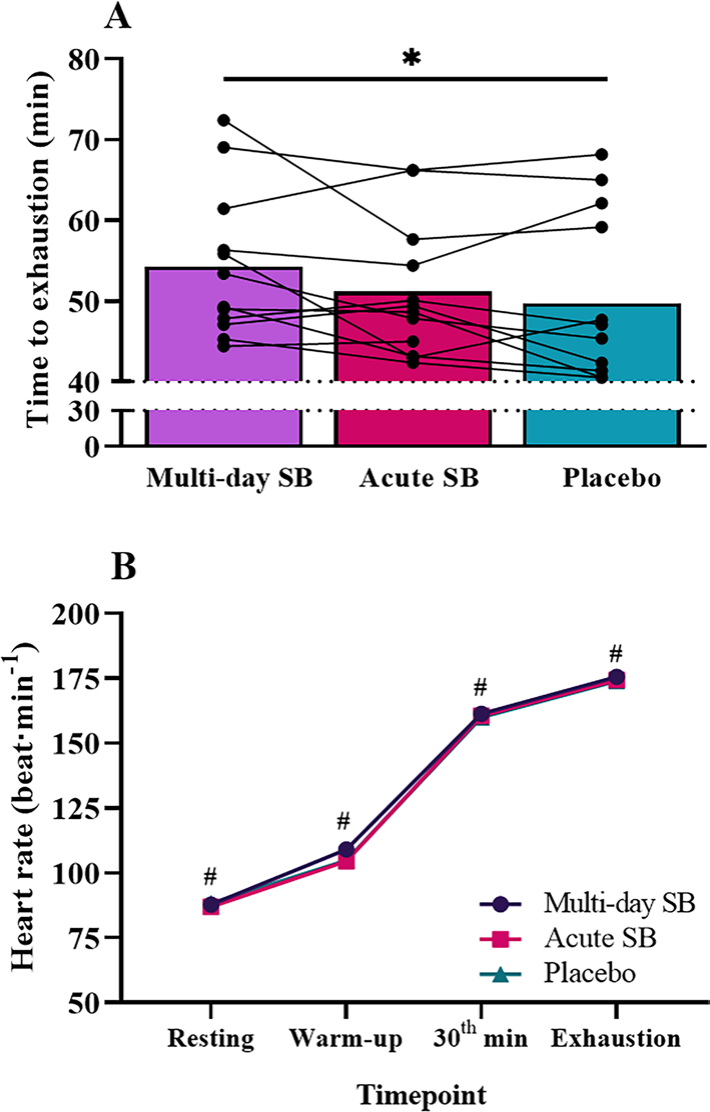
**A** Individual and mean exhaustion times after different bicarbonate supplementation protocols (*n* = 12). **B** Heart rates values during endurance performance test after different bicarbonate supplementation protocols (*n* = 12). *Significant difference between MSB and PLA (*p* < 0.05); ^#^Significant difference between all time points (*p* < 0.05)

Although a significant main effect of the supplementation protocol on time to exhaustion was observed (*F* = 8.13; *p* < 0.05; *η*^2^ = 0.43), the 1% FDR significance threshold (FDR-adjusted *p* value = 0.022) suggests that this effect was a false discovery. Similarly, although Bonferroni post hoc results revealed that MSB (54.27 ± 9.20 min [95% CI 48.43–60.12]) but not ASB (51.15 ± 8.39 min [95% CI 45.82–56.48]) significantly increased time to exhaustion compared with PLA (49.75 ± 10.80 min [95% CI 42.89–56.62]) (*p* < 0.05; Fig. 3A), this increase in MSB was not evident with 1% FDR significance threshold (FDR-adjusted *p* value = 0.040). A significant main effect of exercise was noted on heart rate (*F* = 363.64; *p* < 0.001; *η*^2^ = 0.97), with no main effect of supplementation protocol (*F* = 0.72; *p* > 0.05; *η*^2^ = 0.06) or interaction of protocol and exercise (*F* = 0.38; *p* > 0.05; *η*^2^ = 0.03). Bonferroni post hoc analysis indicated a significant increase in heart rate at each time point (warm-up, at the 30th minute of exercise, and at the time of exhaustion) during the endurance performance test across all conditions (*p* < 0.05) (Fig. 3B).

Dietary records revealed that the total energy intake and macronutrients content of the diets before MSB, ASB, and PLA supplementation were similar (*p* > 0.05; Table 4).

**Table 4:** Comparison of 24-h diet before performance tests (*n* = 12)

Nutrients	Multi-day SB Mean ± SD	Acute SB Mean ± SD	Placebo Mean ± SD	*p*	*F*	*η*^2^
Energy (kcal)	2052 ± 541.2 (1709–2397)	2074 ± 772.5 (1583–2565)	1939 ± 485.9 (1631–2248)	0.554	0.61	0.05
CHO (g)	208.3 ± 81.55 (156.5–260.2)	237.7 ± 114.3 (165.2–310.3)	196.4 ± 69.02 (152.6–240.3)	0.141	2.15	0.16
CHO (%)	41.33 ± 9.22 (35.48–47.19)	46.92 ± 14.29 (37.83–55.99)	41.67 ± 12.05 (34.01–49.32)	0.223	1.61	0.13
Protein (g)	89.82 ± 22.03 (75.82–103.8)	95.35 ± 54.14 (60.95–129.8)	85.11 ± 25.64 (68.11–101.4)	0.615	0.50	0.04
Protein (%)	18.17 ± 3.30 (16.07–20.26)	17.67 ± 5.44 (14.22–21.12)	18.25 ± 5.22 (14.93–21.57)	0.926	0.08	0.01
Fat (g)	92.44 ± 27.60 (74.91–110.0)	87.23 ± 49.39 (55.85–118.6)	88.41 ± 33.09 (67.39–109.4)	0.873	0.14	0.01
Fat (%)	40.33 ± 7.67 (35.46–45.20)	35.25 ± 9.94 (28.94–41.56)	38.06 ± 12.70 (30.01–46.12)	0.377	1.02	0.09

*SB* Sodium Bicarbonate,  *CHO* Carbohydrate, *Mean* ± *SD* Mean ± Standart deviation

### Gastrointestinal responses

Regarding the GI responses, 4 out of 12 participants (33%) in the ASB protocol experienced diarrhea (severity: 5.5 out of 10 points) or bloating (severity: 3 out of 10 points) prior to the endurance performance test. Conversely, in the MSB protocol, 2 out of 12 participants (16.7%) reported bloating (severity: 2.5 out of 10 points) symptoms on each supplementation day, and no symptoms were observed prior to the endurance performance test. Participants reported no GI symptoms either during the supplementation days or prior to the exercise test in PLA protocol.

## Discussion

This is the first study to examine the effects of multi-day low-dose SB ingestion on the performance of high-intensity endurance exercise. Consumption of 0.2 g·kg^−1^·day^−1^ of SB for 4 consecutive days or a single dose of 0.2 g·kg^−1^ ingested acutely 90 min prior to the test, resulted in a significant increase in resting bicarbonate concentrations. However, no significant improvement was noted in high-intensity endurance performance.

Ingestion of both acute and multi-day 0.2 g·kg^−1^·day^−1^ of SB intake increased the buffering capacity as determined by pre-exercise blood bicarbonate and pH concentrations. Resting blood bicarbonate concentrations significantly increased with the consumption of low-dose SB for both consecutive 4 days and acutely 90 min before the exercise test, with an average increase of 2.03 mmol·L^−1^ and 3.74 mmol·L^−1^, respectively. We were unable to compare our findings on the buffering capacity of 0.2 g·kg^−1^·day^−1^ of SB consumption on consecutive days with previous studies that used similar supplementation protocols (Durkalec-Michalski et al. 2018a, b; Durkalec-Michalski, Zawieja, Podgorski, Zawieja, et al., 2018; Joyce et al. 2012), since these studies did not report changes in blood bicarbonate concentrations. However, other studies that investigated the effects of 0.3 g·kg^−1^·day^−1^ or higher doses of SB intake on consecutive days showed that 0.3 g·kg^−1^·day^−1^ (Douroudos et al. 2006; Mueller et al. 2013) and 0.5 g·kg^−1^·day^−1^ (Driller et al. 2012; McNaughton et al. 1999a, b) SB intake for 5 days increased blood bicarbonate and pH concentrations compared to baseline concentration. For example, in two different studies, 0.3 g·kg^−1^·day^−1^ SB intake for 5 consecutive days increased blood bicarbonate concentrations from 26.4 ± 1.8 mmol·L^−1^ to 32.4 ± 1.8 mmol·L^−1^ (Mueller et al. 2013) and from 25.7 ± 2.6 mmol·L^−1^ to 29.8 ± 2.6 mmol·L^−1^ (McNaughton et al. 1999a, b). The findings of this study are consistent with previous studies (Douroudos et al. 2006; Driller et al. 2012; McNaughton et al. 1999a, b; Mueller et al. 2013) demonstrating that multi-day SB intake results in an increase in blood bicarbonate concentrations. It is noteworthy, however, that the observed increase in blood bicarbonate concentration (approximately 2 mmol·L^−1^) in the present study was relatively lower compared to the magnitudes reported in those studies (ranging from 4 to 6 mmol·L^−1^). The lower increase in blood bicarbonate concentrations with both multi-day and single-dose SB intake in the present study is probably due to the lower dose of SB. Conversely, Gough et al., (2017), using the same dose (0.2 g·kg^−1^) as in our study, observed a greater increase in blood bicarbonate concentration after acute ingestion of SB, possibly because they administered SB according to individual time-to-peak blood bicarbonate. Indeed, they (Gough et al. 2017) reported that the individual time-to-peak blood bicarbonate ranged from 40 to 110 min (median 50 min), in contrast to the 90 min duration in the present study. Although one of the limitations of this study is that we did not assess changes in buffering capacity on each day of supplementation, the studies presented above (McNaughton et al. 1999a, b; Mueller et al. 2013) did not show further increases in blood bicarbonate and pH concentrations after the first supplementation day.

Regarding the effects of low-dose SB intake on long-term high-intensity exercise performance, we found that neither a low-dose SB intake for four consecutive days nor acute consumption 90 min before the endurance test was effective in improving high-intensity endurance performance. Although both multi-day (54.27 ± 9.20 min) and acute (51.15 ± 8.39 min) low-dose SB intake resulted in increased exhaustion time compared to placebo (49.75 ± 10.80 min), the difference reached statistical significance only between the MSB and PLA conditions according to post hoc analysis. Nevertheless, it is worth noting that performance tests and the constant load cycling test do exhibit daily fluctuations ranging from 0.9% to 2.0% as previously documented (Hopkins et al. 2001), and that the performance enhancement observed in the current study exceeded the daily variation for both MSB (9.09%) and ASB (2.83%), indicating an improvement in high-intensity endurance performance, particularly after multi days of low-dose SB supplementation. However, the significance did not reach the threshold when using a 1% FDR significance level (FDR-adjusted *p* value = 0.040).

The results of the studies on the effects of acute SB intake on endurance performance are inconclusive; some reported favorable effects (Dalle et al. 2021; Egger et al. 2014; George & MacLaren 1988; N. L. Jones et al. 1977; Leach et al. 2023; Sutton et al. 1981), while others reported no significant effect despite increasing blood bicarbonate and pH or decreasing blood lactate concentration (Freis et al. 2017; McNaughton et al. 1999a, b; Stephens et al. 2002), similar to the findings of the present study. For example, endurance performance at an intensity approximately 4 mmol·L^−1^ lactate threshold was improved by 17% under the alkaline condition (0.2 g·kg^−1^ SB intake prior to exercise) and decreased by 19% under the acidic condition (George & MacLaren 1988). However, despite the same dose administrated in the present study, we were unable to detect such improvements in high-intensity endurance performance, likely due to the participants' training levels (healthy males vs. endurance-trained cyclists) and the specific endurance test protocol utilized (running until exhaustion at 4 mmol·L^−1^ lactate threshold vs. cycling for 30 min at 95% and sprinting at 110% of 4 mmol·L^−1^ lactate threshold until exhaustion). In addition, individual responses in the present study varied significantly, with some individuals experiencing increased performance after SB intake, while others showed no change or even a decrease (Fig. 3A). Moreover, in well-trained cyclists, Egger et al. (2014) reported a 10% improvement in time to exhaustion during an exercise test involving cycling at 110% of anaerobic threshold immediately after training at 95% of anaerobic threshold for 30 min, following ingestion of 0.3 g·kg^−1^ of SB 60 min prior to the test, along with a concurrent increase in blood pH and bicarbonate. Although our study protocol and the training level of the participants align closely with the study by Egger et al. (2014), with the exception of the SB dose, the observed increase in high-intensity endurance performance in the present study (MSB: 9.1% and ASB: 2.8%) remained lower than in the study by Egger et al. (2014) (10%). The observed discrepancy could be attributed to either the dose of SB (0.2 vs. 0.3 g·kg^−1^ SB) or the timing of intake before the exercise test (90 min vs. 60 min). Similarly, previous studies have reported that acute dose of 0.3 g·kg^−1^ SB did improve the 90-s sprint performance by 3% after 3 h of cycling (Dalle et al. 2021) and 16.1 km cycling time trial (TT) performance (Leach et al. 2023). The discrepancy between the results obtained in these studies (Dalle et al. 2021; Leach et al. 2023) and the present study may be explained by differences in the SB dose used (0.3 vs. 0.2 g-kg^−1^ SB) or the specific exercise performance test used (TT vs. TTE). In addition, although not statistically significant, the increase in time to exhaustion for both MSB (9.1%) and ASB (2.83%) exceeded the range of daily variability (0.9 to 2.0%) previously reported (Hopkins et al. 2001). Further study is required to better understand the potential benefits and limitations of low-dose SB supplementation on high-intensity endurance performance.

In the current study we found that 33.3% of participants experienced diarrhea and bloating symptoms with ASB protocol, although a low dose was used. Conversely, only 16.7% of the participants in MSB protocol experienced these side effects as bloating on the pre-test days, but they did not experience any symptoms on the test day. Another study involving CrossFit athletes (Durkalec-Michalski et al. 2018a, b) investigated SB consumption, starting at ¼ of the dose of 0.15 g·kg^−1^ and gradually increasing it up to 0.15 g·kg^−1^ for 10 days. In agreement with our findings they (Durkalec-Michalski et al. 2018a, b) showed that low-dose SB intake over 10 days improved performance in the progressive cycling test and the CrossFit sport-specific performance test without causing GI symptoms. Therefore, the findings of the present study are in agreement with those of previous studies indicating that multi-day low-dose SB consumption avoids GI symptoms.

This study has several strengths, including a double-blind and cross-over design, determination of exercise intensity based on the lactate threshold, ensuring efficient anaerobic metabolism in all participants during the exercise protocol, dietary control, inclusion of trained individuals with cycling race experience, and monitoring of blood pH, bicarbonate, and lactate concentrations using a blood gas analyzer. Limitations of this study are that it did not assess changes in blood bicarbonate concentrations on each consecutive day and individual time-to-peak blood bicarbonate for acute intake to provide a more comprehensive understanding of the effects of low-dose SB supplementation on buffering capacity over time. Another limitation is that the daily variation of the TTE was not examined. Therefore, future studies are recommended to include measurements of blood bicarbonate concentrations on each consecutive day, individual time-to-peak blood bicarbonate for acute intake, and the daily variation of the TTE. Additionally, it is recommended that future studies measure “arterialized” blood samples obtained through the warming of hands using various methods, such as immersion in warm water, heating blanket, and heated air.

In conclusion, this study found that both acute and multi-day low-dose SB intake did not improve high-intensity endurance performance compared to placebo. The lack of a significant increase in endurance performance with acute or multi-day low-dose SB intake may be attributed to various factors, including, the low dose of sodium bicarbonate administered, the lack of assessment of individual time-to-peak blood bicarbonate, and the inherent variability in individual responses. Moreover, in terms of GI discomfort, multi-day consumption of SB was advantageous compared to acute intake, as it did not result in any symptoms on the test day.

### Practical applications

For athletes engaged in endurance sports, SB intake can be considered a potential supplement to enhance performance. However, it is crucial to acknowledge that acute SB intake can potentially lead to GI symptoms, which in turn may impair endurance performance. To address this, a novel SB supplementation protocol involving the administration of 0.1 g·kg^−1^ SB twice daily for four consecutive days (total 0.2 g·kg^−1^·day^−1^) has shown promise improving buffering capacity while minimizing GI symptoms. It is important to note, however, that this dosage may not be sufficiently effective to yield significant performance improvements. Therefore, it is essential to assess individual responses and determine the efficacy of this protocol accordingly. In addition, athletes should collaborate closely with their coaches and medical professionals to determine the most suitable dosage and timing of SB supplementation, tailored specifically to their respective sport and individual needs. By taking a personalized and informed approach, athletes can optimize the potential benefits of SB intake while minimizing any potential drawbacks.

## Data Availability

Data is available from the primary author upon reasonable request.
